# DNA–Liposome Hybrid Carriers for Triggered Cargo Release

**DOI:** 10.1021/acsabm.2c00225

**Published:** 2022-07-15

**Authors:** Kevin N. Baumann, Tim Schröder, Prashanth S. Ciryam, Diana Morzy, Philip Tinnefeld, Tuomas P. J. Knowles, Silvia Hernández-Ainsa

**Affiliations:** † Yusuf Hamied Department of Chemistry, University of Cambridge, Lensfield Road, Cambridge CB2 1EW, U.K.; ‡ Cavendish Laboratory, University of Cambridge, JJ Thomson Avenue, Cambridge CB3 0HE, U.K.; § Department of Chemistry and Center for NanoScience (CeNS), Ludwig-Maximilians-Universität München, Butenandtstr. 5-13, 81377 München, Germany; ∥ Instituto de Nanociencia y Materiales de Aragón, CSIC−Universidad de Zaragoza, Zaragoza 50009, Spain; ⊥ Government of Aragon, ARAID Foundation, Zaragoza 50018, Spain

**Keywords:** DNA nanotechnology, biomimetics, liposome, triggered release, drug delivery

## Abstract

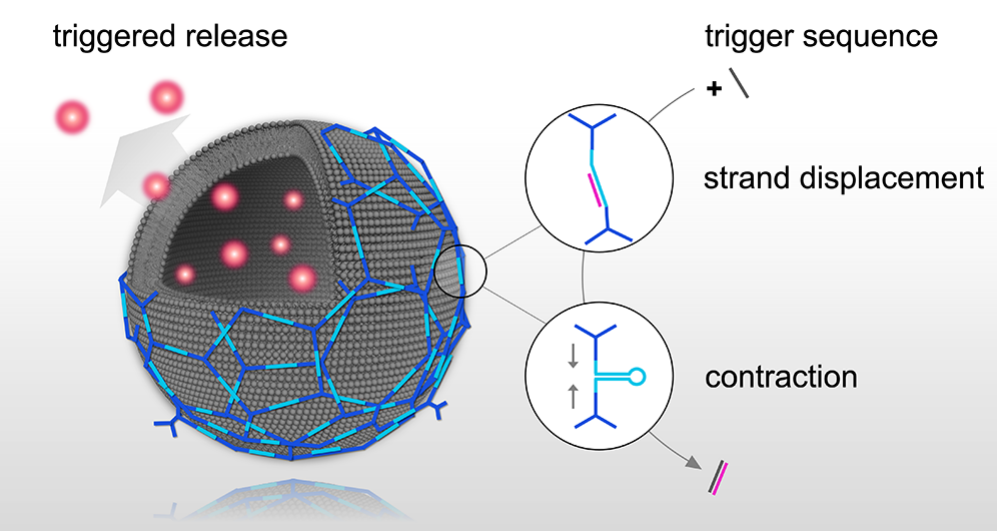

The design of simple and versatile synthetic routes to accomplish triggered-release properties in carriers is of particular interest for drug delivery purposes. In this context, the programmability and adaptability of DNA nanoarchitectures in combination with liposomes have great potential to render biocompatible hybrid carriers for triggered cargo release. We present an approach to form a DNA mesh on large unilamellar liposomes incorporating a stimuli-responsive DNA building block. Upon incubation with a single-stranded DNA trigger sequence, a hairpin closes, and the DNA building block is allowed to self-contract. We demonstrate the actuation of this building block by single-molecule Förster resonance energy transfer (FRET), fluorescence recovery after photobleaching, and fluorescence quenching measurements. By triggering this process, we demonstrate the elevated release of the dye calcein from the DNA–liposome hybrid carriers. Interestingly, the incubation of the doxorubicin-laden active hybrid carrier with HEK293T cells suggests increased cytotoxicity relative to a control carrier without the triggered-release mechanism. In the future, the trigger could be provided by peritumoral nucleic acid sequences and lead to site-selective release of encapsulated chemotherapeutics.

## Introduction

Treatment with many drugs, especially chemotherapeutics, can be associated with severe side effects. Following administration, drug molecules can circulate throughout the bloodstream and can be internalized by a range of cells depending on their rate of metabolism—regardless of whether they are of cancerous or of healthy origin.^1,2^ Another limitation is that small molecules are generally cleared out of the organism rapidly. To address these challenges, the development of larger carrier constructs holds great potential to maximize delivery efficiency, especially if these are targeted.^3−6^ To fulfill a therapeutic effect, however, the drug must become bioavailable by being released from the carrier.^7−9^ This requires precise control over the mechanism and timing of cargo release.^7^

Exogenous triggers, such as electromagnetic radiation, allow the precise timing of trigger deployment, but in many cases require additional intrusion and interference with the organism or the use of highly specialized materials. Moreover, the irradiation of ultraviolet or visible light as trigger types is hampered by the shallow penetration depths in biological tissues.^10−12^ X-rays in particular offer greater penetration and can be used in combination with radiotherapy.^2,13^ In other applications, however, ionizing radiation imposes an additional risk. For these reasons, an entirely autonomous device with release properties dependent on an endogenous trigger event may be a preferable solution.^14^

DNA as a building material has great potential to create a drug delivery vehicle responding to endogenous triggers.^15−18^ The triggered hybridization of DNA can produce forces large enough to facilitate the transition between secondary structures.^19−21^ It has been previously shown that the binding of an aptamer sequence to its target structure can displace prehybridized complementary DNA from the aptamer.^16^ This approach has the advantage that an overexpressed protein can facilitate targeted delivery and selective drug release at the same time. Interestingly, also nucleic acids can be overexpressed by tumor cells and be present at elevated concentrations in the peritumoral environment.^22−24^ The hybridization of these nucleic acids with a corresponding carrier-associated DNA motif could therefore initiate the conversion between secondary structures and thus trigger drug release by direct mechanical interference with the delivery vehicle, such as a lipid vesicle.

Lipid vesicles represent a biocompatible carrier structure that can be produced with high throughput.^25,26^ DNA can be easily anchored to the lipid bilayer mediated by chemically attached hydrophobic moieties.^27−31^ Several studies demonstrate a strong link between the conformation of the DNA structures and the shape or integrity of the lipid bilayer.^27,32,33^ However, many current approaches rely on nonresponsive DNA structures, which impedes exerting controlled activation of lipid vesicle deformation.^32,33^ Triggering the structural activation of liposome-associated DNA building blocks to affect the permeability of the lipid bilayer to specific molecules could offer a potent strategy of generating a drug delivery vehicle with a selective release feature.

**Figure 1:**
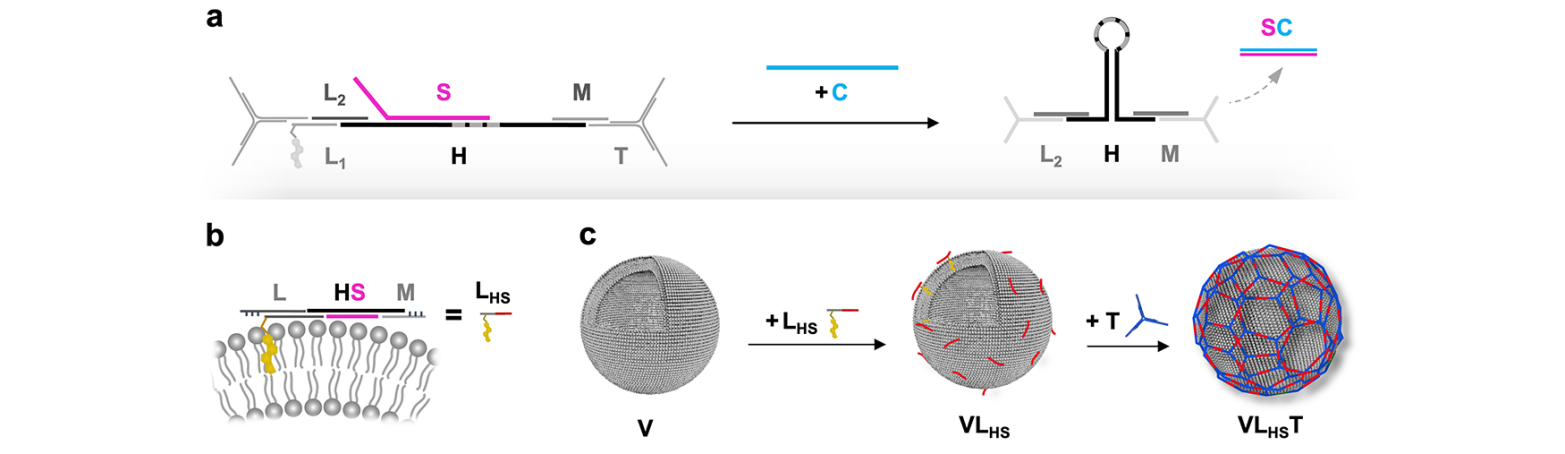
Assembly principle of the active DNA building block and the trigger-responsive DNA–liposomal hybrid nanocarrier. (a) Trigger mechanism of the aDBB. The building block comprises a hairpin H and a preannealed, partially complementary sequence S. Mediated by a toehold at the 5′-terminal of S, a complementary trigger strand C hybridizes with S, allowing H to close. This leads to a contraction of the two opposite ends. (b) The aDBB is first annealed with a cholesterol-TEG-modified linker L (composed of L_2_ and the cholesterol-TEG-labeled L_1_) and a connecting strand M, to render L_HS_. (c) L_HS_ is incubated with large unilamellar POPC vesicles (V) and is anchored to the lipid membranes via the cholesterol-TEG modification (rendering VL_HS_). This allows for polymerization of a triskelion T, which is added in the subsequent step on the surface of the vesicles and hybridizes with M and L_1_ (resulting in the final structure VL_HS_T).

This study presents a method to release small molecules from liposomes by the triggered contraction of “active DNA building blocks” (aDBB). These are arranged on the surface of the liposomes and integrated into a DNA coat, following a modified assembly method of an approach that we described previously.^34^ The addressability of the aDBB leverages the triggered self-hybridization of a DNA hairpin (H), resulting in a contraction of the aDBB. To instate control over the hairpin closure, a spacer strand (S) was preannealed with the hairpin to keep it in an open, or stand-by state (Figure 1a and Supporting information, Section S1, Figure S1). When adding a trigger sequence (C), S can be displaced in a toehold-mediated reaction. The HS DNA duplex (aDBB) was inserted between a cholesterol-triethylene glycol (TEG)-modified linker (L composed of L_1_ and L_2_, Figure 1a) and a DNA triskelion (T) by hybridization via the oligonucleotides L_2_ (part of the linker and partially complementary to H) and M (linking H to T). The cholesterol-TEG modification of L allowed the aDBB to insert into lipid membranes (Figure 1b). In this manner, several of these motifs could be connected on the surface of liposomes in a two-step assembly process: first, the combination of the aDBB, the cholesterol-TEG-labeled L, and M (referred to as L_HS_) was incubated with large unilamellar vesicles (LUVs). Second, the DNA triskelion (T) was added to finalize the coating process (Figure 1c). The element-wise contraction of the DNA building blocks integrated into the DNA coat could alter the permeability of the assembled structures and facilitate the release of encapsulated molecules.

## Results and Discussion

### Triggered DNA Building Block Actuation

**Figure 2:**
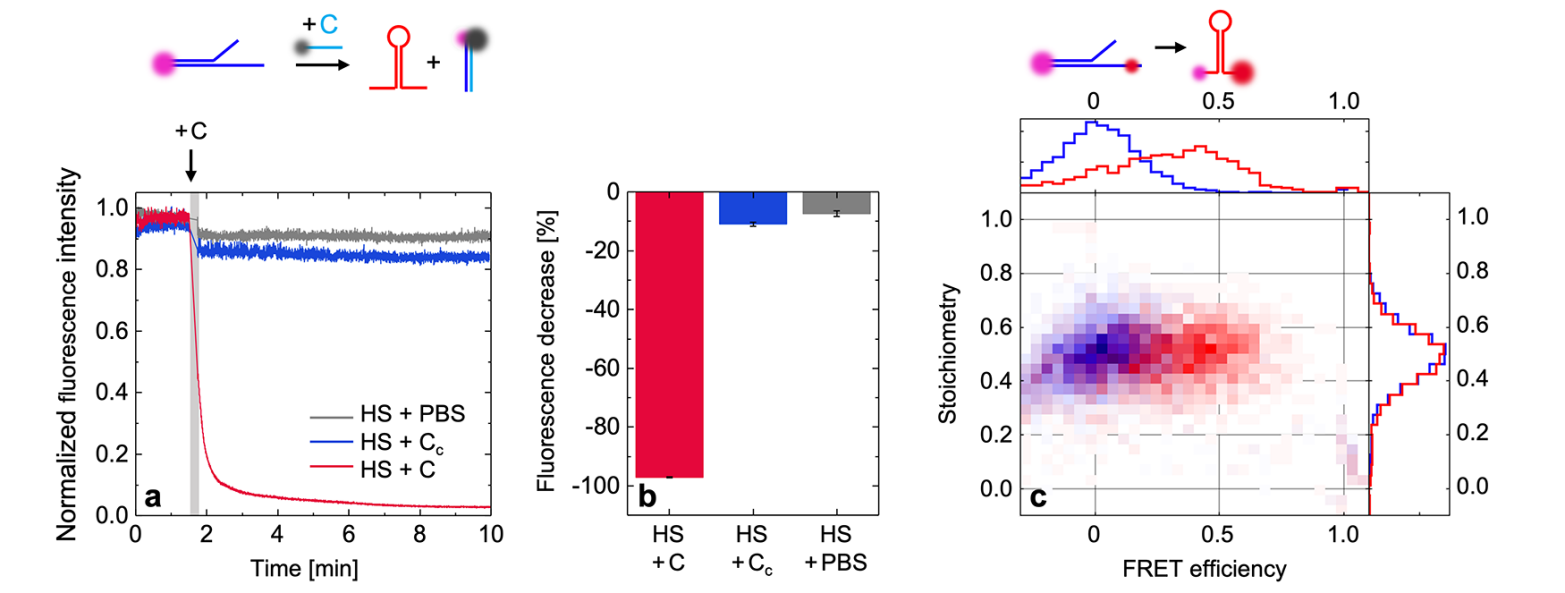
Fluorescence-based assessment of the contraction of the active DNA building block. (a) Addition of C to the aDBB in solution leads to a steep drop of the fluorescence intensity, indicating high quenching efficiencies of the fluorophore placed at the 3′-terminal of S by the quencher placed at the 5′-terminal of C. (b) Strong relative fluorescence decrease suggests the successful displacement of S. A control sequence C_c_ leads to weak unspecific quenching, comparable to the effect caused by dilution with PBS (error bars represent the standard deviation, *n* = 3). (c) Single-molecule FRET measurements support the assumption that the displacement of S results in the contraction of the opposite ends of H due to self-hybridization. This is indicated by an increase in the FRET efficiency between a donor–acceptor pair placed at the two opposite ends of H.

After confirming the assembly of the DNA structures using gel electrophoresis (Supporting information, Section S1 and Figure S1), we probed the ability of the active building block to contract upon stimulation with the trigger sequence C (Figure 1a). Therefore, S was modified with a Cy3 fluorophore (pink sphere, Figure 2a), whereas C was modified with a BlackHole II quencher (black sphere) in the complementary position (Figure 2a). Figure 2a,b shows the fluorescence signal decrease as a result of the hybridization of C and S, indicating the success of the displacement reaction. As a reference, a control sequence C_C_ was added (blue data trace). Here, only a negligible drop of the fluorescence intensity due to stochastic quenching could be observed, comparable to the effect of dilution as simulated by adding pure PBS (gray data trace). Single-molecule Förster resonance energy transfer (FRET) measurements were performed to measure the interdye distance, quantified by the FRET efficiency, upon contraction of the aDBB. To this end, the DNA strands adjacent to H were labeled with a Cy3 fluorophore (pink sphere) as donor molecule (strand L_2_, Figure 2c), and a Cy5 fluorophore (red sphere) as acceptor molecule (strand M, Figure 2c). Before addition of C, the FRET efficiency was zero (Figure 2c), which is reasonable for an estimated donor–acceptor distance of approximately 15 nm and a Förster radius of ca. 5.3 nm^35^ (see Supporting information, Sections S2 and S3, Figure S2, as well as Tables S1 and S2 for further details). After the displacement of S, however, the FRET efficiency shifted to around 45%, which accounts for a distance of approximately 6 nm for this donor–acceptor pair (using γ-corrected values, see also the Methods section).^35−37^ This finding suggests that the displacement of S upon hybridization with C indeed causes a contraction of up to ca. 9 nm of the building block by allowing H to self-hybridize.

### Triggered Actuation of the DNA Building Block on DNA-Coated Liposomes

To confirm the desired integration of the aDBB in the formerly established DNA coat, dynamic light scattering (DLS) and ζ-potential measurements were performed. An increase in the hydrodynamic diameter was observed (Supporting information, Section S4 and Figure S3), accompanied by a decrease of the ζ-potential, when the DNA coats are assembled on the vesicle surfaces. Furthermore, the addition of a detergent led to two populations of size distribution in DLS measurements, denoting detergent-lipid micelles and coexisting DNA assemblies, as we previously described.^34^

**Figure 3:**
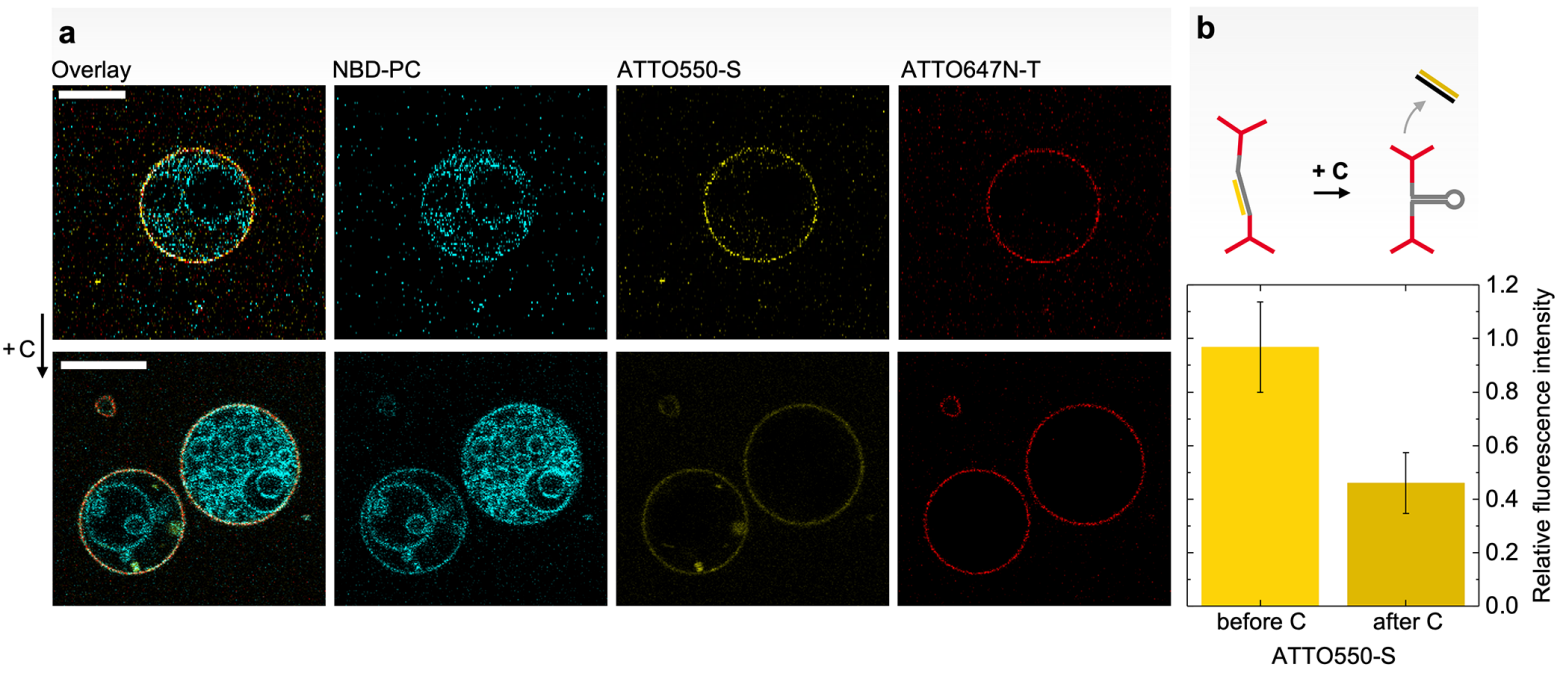
Confocal microscopy of GUVs before and after the addition of the displacement strand C. (a) Confocal micrographs showing the coated GUVs before (top row) and after (bottom row) the addition of C. The cyan channel depicts the NBD-labeled PC lipids in the GUV membrane. The yellow channel shows the ATTO550-labeled S integrated into the DNA coat, and the red channel shows the ATTO647N-coated T. Scale bars: 10 μm. (b) After the addition of C (causing the displacement of S, see sketch), a drop in the fluorescence signal intensity by approximately 50% can be observed (fluorescence intensity relative to the red channel). Error bars represent the standard deviation (*n* = 5).

Next, we sought to gain further insight into the functionality of the aDBB when integrated into the DNA coats on liposomes. To this end, we studied DNA-coated giant unilamellar vesicles (GUVs) by confocal microscopy and fluorescence recovery after photobleaching (FRAP) measurements. Initially, the signal intensities of the ATTO550-labeled S and the ATTO647N-labeled T were analyzed before and after the addition of C (Figure 3a). We observed a drop in the ATTO550 signal intensity by approximately 50% (Figure 3b), which we assigned to C hybridizing with S, and hence displacing S from the DNA coat. The experiments described in the previous section concerning the aDBB suggest that the displacement reaction enables the hairpin to self-hybridize and therefore contract. However, we were not able to visualize large-scale deformation of the DNA coat and the coated GUVs (Figure 3a, bottom row).

**Figure 4:**
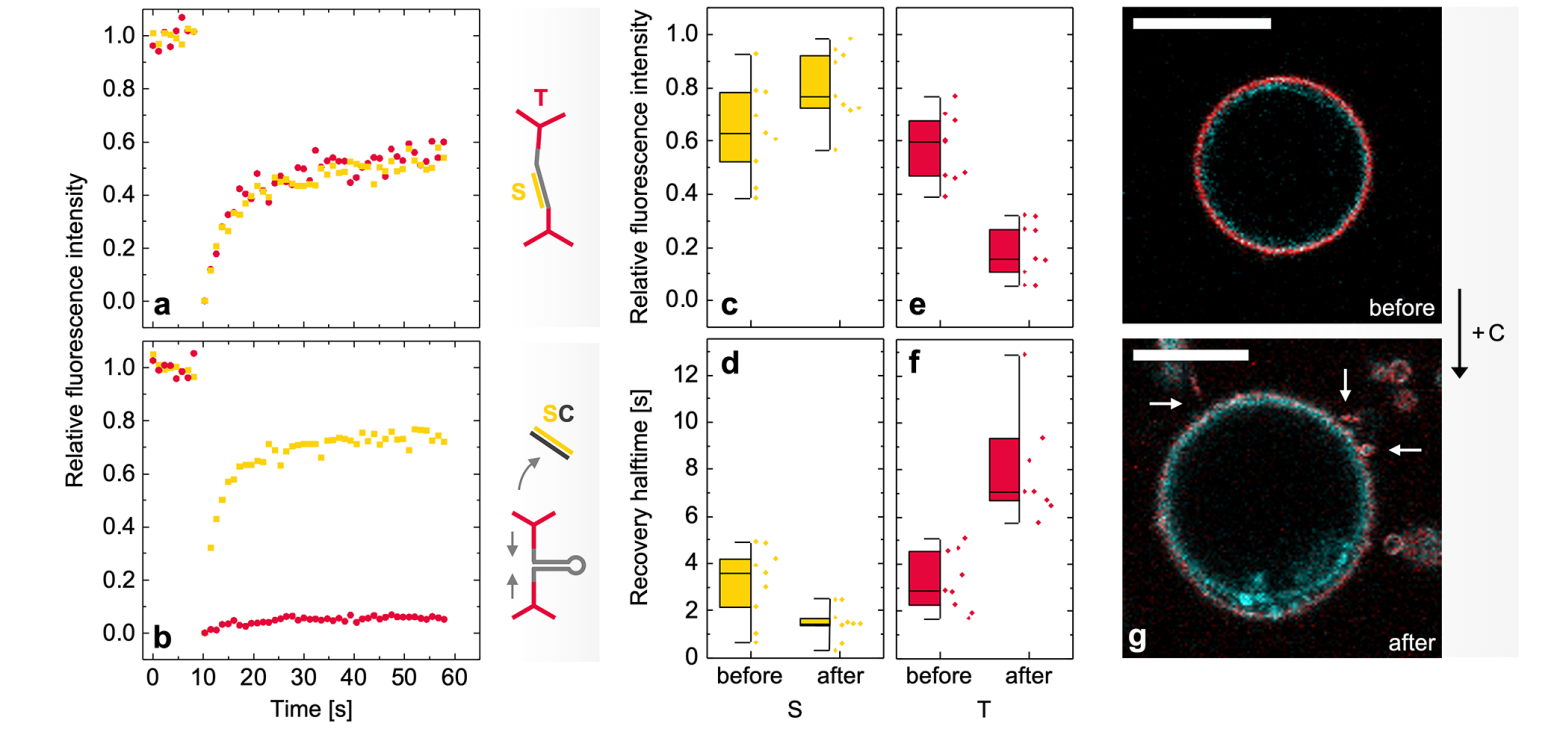
Characterization of DNA-coated GUVs by confocal microscopy and FRAP. (a) Representative FRAP traces recorded on GUVs before the addition of C. S was labeled with ATTO550 (yellow data), and T was labeled with ATTO647N (red data). Both species exhibit similar fluorescence recovery dynamics (described by the recovered fluorescence intensities and recovery halftimes obtained from exponential fitting curves, *n* = 8). (b) After the addition of C, the fluorescence recovery kinetics of S and T split into two populations: S is characterized by increased mobility, while T remains mostly static: the recovered relative fluorescence intensity (c) and the recovery halftime (d) of S is slightly higher. By contrast, a drop in the levels of recovered fluorescence (e) and slow recovery kinetics (f) can be observed for T (box plots show the upper and lower quartiles, as well as the mean, *n* = 8). (g) In the presence of a hyperosmotic pressure, visible deformation and tubulation (see arrows) of the coated GUVs could be observed after the displacement of S had been performed (cyan: NBD-labeled PC lipids, red: ATTO647N-labeled T). Scale bars: 10 μm.

To gain further information on the functional changes of the DNA coat, the fluorescence recovery profiles of the ATTO550-labeled S and the ATTO647N-labeled T were measured before and after adding the trigger sequence C (Figure 4). Before this addition, the two species showed similar recovery kinetics and levels of recovered fluorescence (Figure 4a,c). In comparison to our previously established DNA coats without the aDBB, here, a construct with higher mobility and fewer constraints is present. This may be related to lower polymerization efficiency or to the introduction of more degrees of freedom by structures of larger flexibility (additional sites of ssDNA as knickpoints, refer to Figures 1a and S2, Supporting information).^34^ After adding C, the recovery traces of S and T diverged (Figure 4b) and two populations of recovery behaviors emerge: while S shows faster recovery kinetics and higher recovered fluorescence intensity levels (Figure 4c,d), the recovery of T is represented by slower kinetics and only marginal recovered fluorescence intensity (Figure 4e,f). This corroborates the hypothesis that S is removed by C, which can consequently dissociate from the DNA coat and hence is not restrained in its mobility as in the case of T by the DNA coat anymore (see also Supporting information, Section S5 and Figure S4). These results suggest that the transition of the SC duplex into the solution is limited by steric hindrance imposed by the DNA coat, and possibly magnesium-assisted unspecific adsorption to the lipid bilayer,^38^ which allowed the observation by FRAP with overall reduced signal intensity (relative to the ATTO647N-labeled T by approximately 50%, see also Figure 3b). At the same time, the hairpin can self-hybridize and may stiffen or compact the DNA coat, as indicated by the slow recovery kinetics of T and the reduced post-bleaching fluorescence intensities (Figure 4b,e,f), which exhibit similar properties to the DNA coats we previously described.^34^ While we could not detect visible deformation of the GUVs under isotonic conditions (Figure 3a), we observed membrane budding or tubulation in some cases when the displacement reaction is performed in the presence of a hyperosmotic pressure (Figure 4g).

### Triggered-Release Studies of Calcein Encapsulated by the DNA–Liposome Hybrid Structures

**Figure 5:**
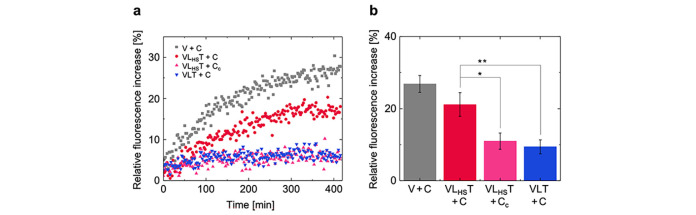
Triggered release of calcein. (a) Relative fluorescence increase of calcein with time displayed by the different structures after C addition. Uncoated LUVs (V + C, in gray), liposomes coated with the aDBB (VL_HS_T + C, in red), and inactive DNA-coated liposomes (VLT + C, in blue). Liposomes coated with the aDBB with a nonhybridizing strand (VL_HS_T + C_c_, in magenta) was added as control. (b) Relative fluorescence increase of calcein exhibited by the structures after C addition at time *t* = 450 min. In comparison to VL_HS_T + C, the calcein release of VLT + C was statistically highly significantly lower (*p* = 0.0060, *n* = 3), similar to the addition of a nonhybridizing C_c_ to VL_HS_T (*p* = 0.0118, *n* = 3). Error bars represent the standard deviation (*n* = 3); one asterisk highlights statistical significance (*p* < 0.05); two asterisks highlights high statistical significance (*p* < 0.01).

As an example of the applicability of the nanoscale DNA–liposome hybrid structures as future triggered-release delivery vehicles, we investigated potential changes in the permeability of entrapped small molecules. Therefore, the lipid films were rehydrated in a calcein-containing solution to generate calcein-laden LUVs (see the Methods section). The leakage of calcein from the LUVs is expressed by an increase in the fluorescence intensity, as the consequent dilution of the fluorophore results in a loss of self-quenching, which only occurs at high concentrations. Initially, we measured the passive leakage of calcein from pure liposomes (V) in comparison to the coated structures VL_HS_T and VLT (DNA-coated vesicles without the aDBB; Supporting information, Section S6 and Figure S5). Interestingly, the addition of the DNA coats greatly reduced the leakage of calcein over the course of the measurement. To study the active release, the calcein fluorescence was measured after the trigger sequence C was added to the samples V, VL_HS_T, and VLT (Figure 5a) and compared to the values before C supplementation (Figure 5b). The relative increase in fluorescence intensity displayed by the active carrier (VL_HS_T) upon C supplementation (red data, Figure 5b) was significantly higher (*p* = 0.0060, *n* = 3) than the value observed in the case of VLT (blue data, Figure 5b) denoting elevated permeability of the entrapped calcein triggered by the addition of the displacement strand C. The relative enhancement in fluorescence exhibited by the active carrier is close to the increase displayed by pure POPC liposomes (gray data, Figure 5b). As observed, uncoated liposomes showed a stronger response to the osmotic change induced by the addition of C than DNA-coated controls (VL_HS_T + C_C_, VLT + C). In addition, we performed a control experiment where an inactive sequence C_C_ was incubated with VL_HS_T. In comparison to C (red data, Figure 5b), the calcein released by C_C_ (magenta data, Figure 5b) was statistically significantly lower (*p* = 0.0118, *n* = 3). C_C_ did not cause a similar permeability benefit, thus excluding the possibility that the release in the active carrier was only an effect of osmotic changes.

### Cytotoxicity of Doxorubicin-Laden DNA–Liposome Hybrid Structures

**Figure 6:**
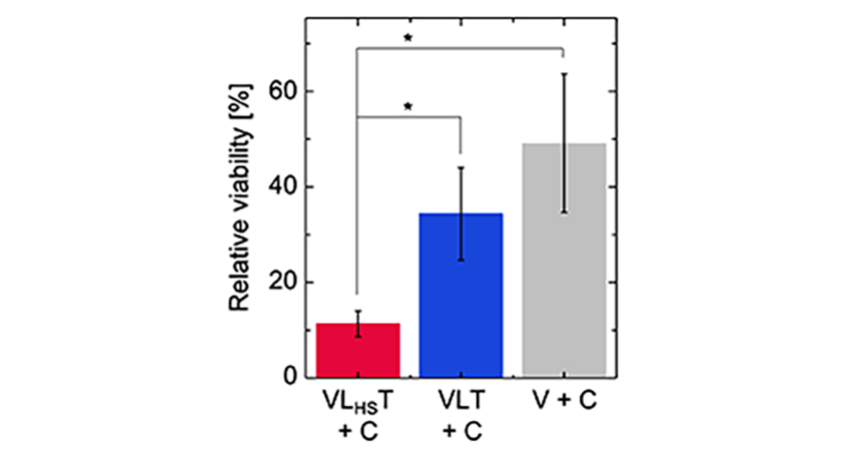
Cell viability displayed by doxorubicin-laden vesicles upon addition of C. VL_HS_T: DNA-coated vesicles with the aDBB; VLT: nonresponsive DNA-coated vesicles; V: uncoated vesicles. In comparison to VLT, the cytotoxic response is statistically significantly increased (*p* = 0.0159, *n* = 3). The cytotoxic effect of uncoated vesicles (V) is comparable to VLT, but statistically significantly different from VL_HS_T + C (*p* = 0.0111, *n* = 3). Error bars represent the standard deviation (*n* = 3); the asterisk highlights statistical significance (*p* < 0.05).

To further explore controlled release and drug delivery applications, we investigated the cytotoxicity displayed by the hybrid structures loaded with the widely used chemotherapeutic doxorubicin (DOX).^39−41^ As a model culture, HEK293T cells were incubated with DOX-laden responsive (VL_HS_T), and nonresponsive (VLT) hybrid structures, as well as uncoated LUVs (V). The trigger strand C was added to the DOX-laden carriers, whereby only the VL_HS_T design is expected to respond to the trigger and increase the permeability to DOX. After the incubation time, the cytotoxic effect of the two carriers was estimated using a luciferase viability assay. Figure 6 shows that upon addition of C, the toxicity of the trigger-responsive carrier design (VL_HS_T) is significantly increased with respect to the nonresponsive VLT design (*p* = 0.0159, *n* = 3), which we assign to the enhanced permeability of the vesicle promoted by the activation of the aDBB. The cytotoxic effect of uncoated vesicles (V) is comparable to VLT, but statistically significantly different from VL_HS_T + C (*p* = 0.0111, *n* = 3).

## Conclusions

In conclusion, in this study, we demonstrate an approach to add release functionality to DNA-coated vesicles. We provide evidence that the triggered closure of a DNA hairpin can influence the DNA coat and lipid membrane properties. This effect is likely to originate from the contraction of the hairpin following its self-hybridization. This strategy can be applied to triggered-release purposes, which we evidence by the release of dye molecules and the enhanced cytotoxicity induced by the DOX-laden trigger-responsive coated liposomes. In the future, the presented method may inspire similar approaches with maximized control of the deformation of liposomes. We believe that upon adequate optimization using nonimmunogenic oligonucleotides^42^ and precise lipid nature and composition,^43^ a system like this can have significant potential for nanotherapeutic applications as it allows molecules to be transported and released only when a trigger is present. This could be exploited, for instance, in the proximity of tumors, where the bioavailability of enclosed molecules can be increased by triggering the release through the interaction with pathophysiologically overexpressed biomolecules and the carriers.^3,44−48^

## Methods

### Folding of the Active DNA Building Block

The aDBB was folded in two steps. First, the strands S and H were hybridized using a custom thermal protocol: in 1x PBS (pH = 7.4), or in an aqueous solution of 75 mM Na_2_HPO_4_, (pH = 7.4) for the leakage measurements, 6 μM of the two oligonucleotides was suspended and heated to 85 °C for 5 min, before cooling to room temperature at a rate of −0.5 °C per minute. Eventually, the samples were kept at 4 °C. In the second step, the S–H duplex was incubated with L (separately annealed following our previously published protocol^34^) and the strand M. All oligonucleotides were purchased from Integrated DNA Technologies (IDT). The sequences of all involved oligonucleotide sequences can be reviewed in Supporting information Table S1.

### Gel Electrophoresis

Polyacrylamide gel electrophoresis (PAGE) was performed to evaluate the folding success of the DNA structures. The gels were prepared with 10% polyacrylamide in 11 mM MgCl_2_ buffered at pH = 8.3 with 0.5x TBE and run for 60 min at 100 V, immersed in a solution containing 11 mM MgCl_2_ buffered at pH = 8.3 with 0.5x TBE.

### Fluorescence Quenching Measurements

The efficacy of the toehold-mediated displacement reaction in removing the spacer strand was further verified by measuring fluorescence quenching when hybridizing with the displacement strand. To this end, C was labeled with an Iowa Black-quencher in the 5′-terminal and S with a Cy3 fluorophore in the 3′-terminal (purchased from IDT). The degree of fluorescence quenching upon addition of the displacement strand correlates with the amount of displaced spacer. The aDBB was studied at a concentration of 2 μM; the displacement strand was added at 2× excess (to increase the displacement success, following PAGE results). As a control, a nonhybridizing sequence C_C_ was labeled with an Iowa Black-quencher and added under the same concentration conditions. As a further control, PBS was added at the same volume as C_C_ to compare the stochastic quenching originating from the addition of C_C_ to the fluorescence reduction originating from dilution.

### Single-Molecule FRET Measurements

All oligonucleotides were purchased at a concentration of 100 μM from IDT in nuclease- and salt-free buffer for the single-molecule FRET measurements. For assembling of the aDBB, the oligonucleotides (sequences used for FRET are also indicated in Table S1) were mixed according to Table S2 in aliquots of 20 μL in a solution containing 12.5 mM MgCl_2_ buffered with 10x TAE. The sample solution was subjected to the thermal protocol summarized in the Folding of the Active DNA Building Block section. Finally, the mixture was purified with a 4% 1x TBE agarose gel, which was run for 30 min at 160 V in the same buffer. The slowest band (formed by the desired product) was cut out, and the structure was extracted by squeezing the cut-out between cover slides. Prior to the single-molecule FRET measurements, the samples were diluted in 1x PBS to achieve a concentration of 100 pM. The displacement reaction was performed by adding D at a concentration of 8 μM. The single-molecule FRET experiments by pulsed interleaved excitation (PIE)^49^ were carried out with a custom-built confocal microscope. To this end, the DNA was placed in custom-built 60 μL imaging chambers. The fluorescent donor molecules were excited by a pulsed diode laser (LDH-P- FA-530B, PicoQuant, Germany), at 532 nm operated with a 20 MHz repetition rate. The excitation intensity was adjusted to 30 μW. The fluorescent acceptor molecules were excited by a pulsed diode laser (LDH-D-C-640, PicoQuant), at 639 nm operated with a 20 MHz repetition rate. The excitation intensity of the sample was adjusted to 30 μW. The laser pulses were separated by 25 ns by a multichannel picosecond diode laser driver (PDL 828 “Sepia II”, PicoQuant) with an oscillator module (SOM 828, PicoQuant). The lasers were coupled into a single-mode fiber (P3-488PM-FC, Thorlabs) to obtain a Gaussian beam profile and overlaying laser beams. Circular polarized light was obtained by a linear polarizer (LPVISE100-A, Thorlabs) and a quarter-wave plate (AQWP05M- 600, Thorlabs). The laser light was guided into the epi-illuminated confocal microscope (Olympus IX71, Olympus, Japan) by dual-edge beam splitter (z532/633, AHF Analysentechnik AG, Germany) focused by an oil immersion objective (UPLSAPO100XO, NA 1.40, Olympus). The emitted fluorescence was collected through the objective and spatially filtered using a pinhole with a 50 μm diameter and spectrally split into donor and acceptor channel by a single-edge dichroic mirror (640DCXR, AHF Analysentechnik AG). Fluorescence emission was filtered (donor: Brightline HC582/75 (AHF Analysentechnik AG) and RazorEdge LP 532 (Laser 2000, Germany); acceptor: Shortpass 750 (AHF Analysentechnik AG) and RazorEdge LP 647 (Laser 2000)) and focused on avalanche photodiodes (SPCM-AQRH-14-TR, Excelitas Technologies). The detector outputs were recorded by a time-correlated single-photon counting module (HydraHarp 400, PicoQuant). The setup was controlled by a commercial software package (SymPhoTime64, Picoquant). Data analysis was performed using the “PAM” software package as described by Schrimpf et al.^50^ Single-molecule events were identified using a two-channel APBS algorithm with a threshold of 10 photons per time window of 500 μs and a minimum photon count of 30. γ correction was performed using the protocol published by Hellenkamp et al.^37^ To remove donor or acceptor-only events, the ALEX-2CDE filter was applied using an upper threshold of 15.^51^

### Fabrication of DNA–Liposome Hybrid Carriers

LUVs were prepared by extrusion of a 2 mM 1-palmitoyl- 2-oleoyl-sn-glycero-3-phosphocholine (POPC, purchased as powder from Sigma-Aldrich and stored dissolved in chloroform) lipid suspension. The lipids were suspended in 1x PBS, sonicated, and extruded through a 200 nm pore size membrane (see Supporting information, Section S7, Figure S6).

The coated liposomes were prepared in aliquots of 100 μL. The LUVs (50 μL) were incubated with approximately 1 μM aDBB (preannealed with the cholesterol-labeled linker) overnight at room temperature (diluting the POPC lipids to 1.2 mM), rendering VL_HS_. Subsequently, the triskelion was added to obtain a final concentration of approximately 550 nM and incubated with VL at 4 °C for 50 min (VL_HS_ pre-tempered). This led to a dilution of the linker to approximately 830 nM. Due to the addition of the DNA, the liposomes were effectively diluted by half.

### Characterization by Dynamic Light Scattering and ζ-Potential Measurements

Hydrodynamic diameters and ζ-potentials (Supporting information, Section S4, Figure S3) were measured with a ZetaSizer Nano ZSP by Malvern Panalytics. All samples were measured in disposable cuvettes at a final lipid concentration of 1 mM in PBS. To measure the ζ-potential, the samples were additionally diluted 1:8 in PBS.

### Confocal Microscopy and FRAP Measurements

Confocal microscopy was performed to assess the ability of the DNA structures to coat and deform the membranes of GUVs. Thus, GUVs (including 200:1 (w/w) NBD-labeled PC) were generated by electroformation, using the protocol described in our previous study.^34^ The linker was annealed with the active hairpin (pre-assembled with the spacer strand as described above) and subsequently added at a concentration of 200 nM and incubated for 2 h. Finally, the triskelion was added at room temperature and incubated for another hour (2 μL of 6 μM T). The spacer strand was purchased with a 5′-ATTO550 modification from IDT. All three arms of the triskelion were labeled with an ATTO647N fluorophore at the 5′-terminal (purchased by IDT). Imaging was conducted using an Olympus F1200 microscope and a 60× oil immersion objective. The samples were illuminated with 488 nm (NBD-labeled PC lipids to visualize the lipid membranes), 535 nm (ATTO550-labeled spacer), and 635 nm (ATTO647N-labeled triskelion) lasers in line-sequential acquisition. The coated GUVs were treated with the displacement strand C for 3 h to remove the spacer strand and allow the hairpin to close to achieve deformation of the vesicles. Before and after this displacement reaction, FRAP analysis was performed to evaluate the diffusion properties of the spacer and triskelion (as an indicator of the extent of DNA polymerization) by bleaching a 3 μm large area with the 535 and 635 nm lasers into the DNA coats. The hyperosmotic pressure was induced by performing the displacement reaction in a solution with an approximately 10% higher osmolarity (by adjusting the glucose concentration).

### Calcein Release Experiments

POPC lipid films were rehydrated (day 1) in a 60 mM calcein solution diluted in 75 mM Na_2_HPO_4_ (pH = 7.4). The LUVs were purified from free dye by gel filtration using Sephadex G50. Subsequently, the DNA was added as described in the above section, outlining the DLS experiments. The modified linker in combination with the active hairpin (VL_HS_) was added on day 1, as well as a version with the inactive hairpin, by omitting S (VL_H_). On day 2, the T1 triskelion was added to both VL_HS_ and VL_H_ and incubated for 1 h at 4 °C. Finally, the displacement strand S was added to all samples (to account for dilution effects in the non-trigger-responsive samples), and the fluorescence intensity was recorded over time with a ClarioStar Plus plate reader (BMG Labtech, Germany) excitation wavelength of 488 ± 15 nm, and emission was recorded at 515 ± 20 nm at room temperature. As a control, C_c_ was added (altered sequence to prevent hybridization with the spacer strand S). At the end of the acquisition time, 1% Triton X-100 was added to disrupt the vesicles and record the maximal achievable fluorescence intensity. Relative fluorescence intensity was calculated as described in Section S6 of the Supporting information.

### Incubation of the Doxorubicin-Laden DNA–Liposome Hybrid Carriers with HEK293T Cells

The coated liposomes were fabricated to trap doxorubicin (DOX) and incubated with HEK293T cells. DOX is a widely used anticancer drug and imposes toxicity toward cells by inducing DNA strand breaks.^52,53^ Coated liposomes were prepared by rehydrating POPC lipids in a solution of 5 mg/mL DOX in sterile 1x PBS following the protocol summarized in the previous section (Calcein Release Experiments). DOX was purchased as a powder from Stratech Scientific, U.K. A total of 2500 cells were seeded per well of a 96-well plate and covered by 100 μL of DMEM supplemented with 10% fetal bovine serum and glutamax. The cells were environmentally controlled at 37 °C and 5% CO_2_ incubated for 3 days to allow adherence and confluence. On the third day, incubation with the coated liposomes was performed. Alongside the VL_HS_T carriers, VLT and V were incubated. For each sample, three wells were prepared for incubation by adding 90 μL of sample solution per well for approximately 30 min. Afterward, 5 μL of the displacement strand D, present in a 30 μM solution in 1x PBS was added. To three separate wells, 95 μL of 1x PBS only was added as a nontoxic control. After an incubation time of approximately 3.5 h, the supernatant was removed from each well and 30 μL of trypsin solution was added to detach the cells. After 2 min of trypsinization, the reaction was blocked by adding 100 μL of fresh culture medium. The cells were then transferred into centrifugation tubes and spun down for 5 min at 300 rcf. Finally, the supernatant was removed and the cell pellets were resuspended in 1x PBS and counted using an automated cell counter (Countess, Thermo Scientific) to adjust for deviating cell numbers for the subsequent viability assay. To assess the viability of the HEK293T cells after the treatment, an ATP-sensitive luciferase bioluminescence assay was performed with CellTiter Glo (Promega). To perform the assay, 100 μL of the cells in 1x PBS was pipetted into wells of a black 96-well plate (Greiner, Austria) at an approximate concentration of 10 000 per μL. The luciferase buffer (30 μL) was added to each well and incubated for 10 min at 37 °C. The luminescence emission was analyzed using a ClarioStar Plus plate reader. To calculate the relative cell viability, the luminescence values (*I*) were normalized to the nontoxic control (HEK293T + PBS, *I*_max_)
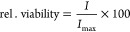


## Supporting Information Available

DNA sequence information; gel electrophoresis results; additional FRAP data; and additional leakage information (PDF)

## Notes

A preprint version of this manuscript is available on ChemRxiv: K.N.B., T.S., P.S.C., D.M., P.T., T.P.J.K., and S.H.-A.; DNA-Liposome Hybrid Carriers for Triggered Cargo Release; https://doi.org/10.26434/chemrxiv-2022-x731g-v2.

## Abbreviations

DNA: deoxyribonucleic acid
RNA: ribonucleic acid
dsDNA: double-stranded deoxyribonucleic acid
ssDNA: single-stranded deoxyribonucleic acid
S: spacer strand
H: DNA hairpin
HS: duplex of H and S
T: triskelion
C: trigger sequence complementary to S
C_C_: control sequence not complementary to S
V: large unilamellar POPC vesicles
aDBB: active DNA building block
VL_HS_: intermediate incubation step of vesicles and the cholesterol-modified aDBB
VL_HS_T: trigger-responsively coated V
VLT: DNA-coated V without trigger mechanism
