# Supplementary material for: DNA–Liposome Hybrid Carriers for Triggered Cargo Release
Source: ACS Appl Bio Mater. 2022 Jul 15;5(8):3713–21. doi: 10.1021/acsabm.2c00225 (PMC9382633; doi:10.1021/acsabm.2c00225)
Supplement: Supplementary file 1 — mt2c00225_si_001.pdf [file mt2c00225_si_001.pdf]

Supporting Information

DNA-Liposome Hybrid Carriers for Triggered Cargo Release

Kevin N. Baumann[1,2†], Tim Schröder[3], Prashanth S. Ciryam[1‡], Diana Morzy[2§], Philip Tinnefeld[3], Tuomas P. J. Knowles[1,2], Silvia Hernández-Ainsa[4,5]**

AUTHOR ADDRESSES

1 Yusuf Hamied Department of Chemistry, University of Cambridge, Lensfield Road, Cambridge CB2 1EW, United Kingdom

2 Cavendish Laboratory, University of Cambridge, JJ Thomson Avenue, Cambridge CB3 0HE, United Kingdom

3 Department of Chemistry and Center for NanoScience (CeNS), Ludwig-Maximilians-Universität München, Butenandtstr. 5-13, 81377, München, Germany

4 Instituto de Nanociencia y Materiales de Aragón, CSIC–Universidad de Zaragoza, Zaragoza 50009, Spain

5 ARAID Foundation, Government of Aragon, Zaragoza 50018, Spain

* correspondence to silviamh83@unizar.es, tpjk2@cam.ac.uk

Table of contents

S1. Characterization of the DNA nanostructures by Polyacrylamide Gel-Electrophoresis

S2. Triggered contraction and DNA Sequence Information

S3. Characterization of the DNA Nanostructures by Single-Molecule FRET Measurements

S4. Characterization of the DNA-Liposome Hybrid Carriers

S5. Confocal Microscopy Imaging of DNA-Coated GUVs

S6. Calcein Release Experiments

S7. Liposome Preparation

S1. Characterization of the DNA nanostructures by Polyacrylamide Gel-Electrophoresis

The folding of the DNA nanostructures L, H, and the active DNA building block (aDBB) were examined using 10% polyacrylamide gel-electrophoresis (Figure S2). Apparent from the band in lane 3 in Figure S2a is that natively the hairpin is preferably arranged into superstructures. The hairpin subjected to a thermal protocol (heating to 85 °C for 5 min, then cooling to 25 °C at a rate of -1 °C/min, eventually holding at 4 °C) increases the yield of singular structures, rendering a single band (lane 1 in Figure S2b). The gel in Figure S2b confirms that the hairpin H associates with S (lane 2), which is expressed by the shift of the band in comparison to H alone (lane 1). The aDBB (Figure S1a, lane 6) was assembled in two steps from the HS duplex (lane 4): first L (lane 5) was added, and then in addition M (lane 6), showing a further upshift of the band in the gel (compare mobilities between lanes 4, 5, and 6). These upshifts indicate successful hybridization of HS with both M and L and the formation of the aDBB.

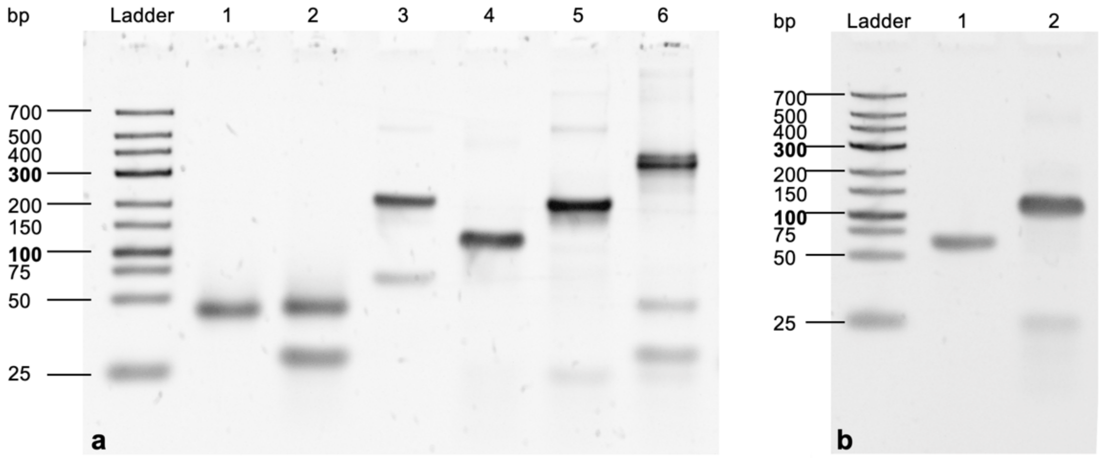

Figure S1. Polyacrylamide gel-electrophoresis following the assembly of the aDBB. (a) The lanes contain the linker L (1), the linker together with the connecting strand M (2), the hairpin (3), the hairpin with the spacer S (yielding aDBB, 4), HS and L (5), and L_{HS} (aDBB + L + M, 6). The two bands in lane (2) confirm that L and M do not hybridise or form other superstructures. Together with HS (lane 6), the aDBB is formed, indicated by the band positioned at the height of the 300 bp mark. Two bands of weaker intensity are found at the position of L and M. Visible in lane 3 is that H preferably forms superstructures. (b) Subjected to a thermal programme (as outlined above), a single band at a position in the gel which matches the number of nt of H can be found (lane 1). The band was run next to HS (lane 2) for comparison. The shift of the band denoting HS compared to H confirms the hybridisation of the two sequences.

S2. Triggered contraction and DNA Sequence Information

The oligonucleotide sequences are gathered in the table below (Table S1). To estimate the maximum possible contraction that the triggered hybridization of H can cause, we assumed a distance of 0.34 nm for each base pair of a double-stranded DNA sequence.[1] The single-stranded part of the hairpin left after hybridizing with S was estimated to account for a coil with an approximate diameter of 4 nm.[2] Prior to the addition of C, the aDBB would therefore have the length of 32 bp (H-S complementary part) * 0.34 nm/bp + 4 nm = 14.9 nm. This distance equals the theoretical distance between donor (placed on L_{2FRET}) and acceptor (placed on M_{FRET}) as used

for the single-molecule FRET measurements. The hairpin before and after subtraction together with the sequences is visualized below (Figure S2).

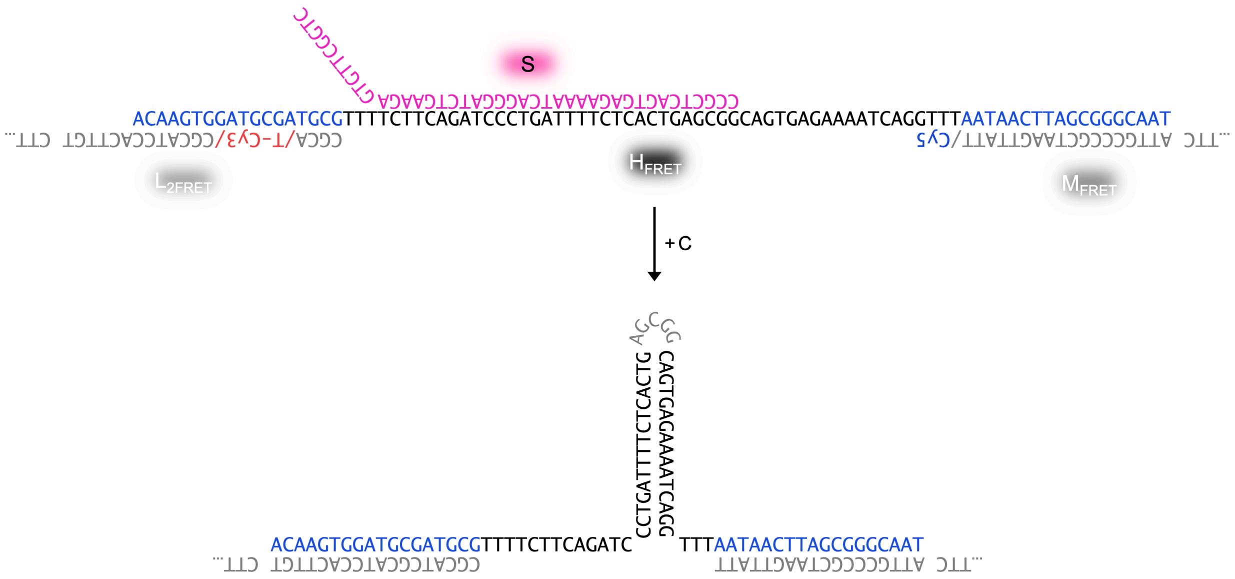

Figure S2. Triggered contraction mechanism of the aDBB. The building block initially comprises a hairpin H and a pre-annealed, partially complementary sequence S (magenta). The aDBB further hybridizes with the strand L_2 and M. The blue part of the H sequence shows the complementary part of H and L_2 or M, respectively (mediated by a toehold at the 5'-terminal of S, a complementary trigger strand C hybridizes with S, allowing H to close. This leads to a contraction of the two opposite ends. Note that for the FRET studies the length of the self-hybridizing part of H_{FRET} remained the same as the original design H even though the sequences of the sticky ends pairing with M_{FRET} and L_{2FRET} differ. For simplicity, the sequences used for aDBB in the FRET studies were designed with no single-stranded overhangs of M_{FRET} and L_{2FRET} (which are present in the original design to allow for the subsequent hybridization with T and the formation of $VL_{HS}T$).

Table S1. Oligonucleotide sequences of all involved DNA strands. Functionalizations (when applicable, as declared in text) are indicated in the sequences accordingly.

Name	Oligonucleotide sequence
H	5’-ACAAGTGGATGCGATGCG<br>TTTTCTTCAGATCCCTGATTTTCTCACTGAGCGG<br>CAGTGAGAAAATCAGGTTT AATAACTTAGCGGGCAAT-3’
H_{FRET}*	5’-GCTCGGTACCCGGGGATCCT<br>TTTTCTTCAGATCCCTGATTTTCTCACTGAGCGG<br>CAGTGAGAAAATCAGGTTT AAGCTTGGCACTGGCCGTCG-3’
S	5’-(ATTO550)/CCGCTCAGTGAGAAAATCAGG GATCTGAAGAGT<br>GTTCGGTC-3’
C	5’-GACCGAACAC<br>TCTTCAGATCCCTGATTTTCTCACTGAGCGGT(/BQII)-3’
C_C	5’-TCAAGAGCACAATGCAGAATCATGCATCGTACCCTGACGGAT-3’
L_1	5’-TCTCTTTGGATAATCTTCCCTCTGCTGCCGCTCAAG/CholTEG -3’
L_2	5’-CGCATCGCATCCACTTGTCTTGAGCGGCAGCAGAGG-3’
$L2_{FRET}$*	5’-AGGA/T-Cy3/CCCCGGGTACCGAGC-3’
M	5’-TCTCTTTGGATAATCTTCATTGCCCGCTAAGTTATT-3’
M_{FRET}*	5’-CGACGGCCAGTGCCAAGCTT/Cy5/-3’
T_1	5’-(ATTO647N)/GAAGATTATCCAAAGAGATCCGAGTTGTCTT<br>GTACAT CCTGAAGATACATCAAGC-3’
T_2	5’-GAAGATTATCCAAAGAGAGGTAGGCTAGTATCTGTGT<br>TGTACAAGACAACTCGGA-3’
T_3	5’-GAAGATTATCCAAAGAGA<br>GCTTGATGTATCTTCAGGTCACAGATACTAGCCTACC-3’

*The sequence H_{FRET} represents an adaption of H where the parts hybridizing with L_2 and M were modified to allow for hybridization with L_{2FRET} and M_{FRET}. Length and sequences of the self-complementary part were not changed. The part of the sequence that had been changed to obtain H_{FRET} is colored in grey.

S3. Characterization of the DNA Nanostructures by Single-Molecule FRET Measurements

The concentrations of the individual DNA strands used for single-molecule FRET experiments are summarized in Table S2. The corresponding sequences can be found in the Table S1.

Table S2. Concentrations of the DNA strands used for single-molecule FRET measurements probing the triggered contraction of the aDBB.

Sequence name	Concentration [μM]
H_{FRET}	10
S	40
Cy3-L_{2FRET}	20
Cy5-M_{FRET}	20

S4. Characterization of the DNA-Liposome Hybrid Carriers

The assembly of the DNA coats on 200 nm LUVs was tracked observing the change of the hydrodynamic diameter using dynamic light scattering and zeta potential measurements (Figure S3). After the incubation with the aDBB, the average particle size increases by about 30 nm. An additional 20 nm can be observed when finalizing the coating process by the addition of T. At the same time the zeta potential drops to approximately -32.4 ± 2.3 mV for the completed DNA coat assembly ($VL_{HS}T$). As previously reported, the addition of Triton X-100 (TX100) to LUVs coated with the linker and triskelion (without the aDBB) solubilizes the liposomes while leaving connected or partially connected DNA assemblies.[3] In the size distribution graph this process was expressed by the appearance of two peaks: the first peak centered at around 10 nm denotes detergent-lipid micelles, while the second peak represents the DNA assemblies of linker (L) and

triskelion (T) and can be found at the original size position (diameter in intensity around 250 nm). Incompletely coated LUVs were completely solubilized without the appearance of DNA assemblies. We therefore added TX100 to the DNA-liposome hybrid carrier $VL_{HS}T$ to assess the quality of the DNA coats. Indeed, a double-peak size distribution could be observed by DLS after TX100 was supplemented (Figure S3). After displacing S by adding C, the same experiment was performed. Similarly, the detergent did not completely disassemble the structures, indicated by the presence of the double-peak size distribution.

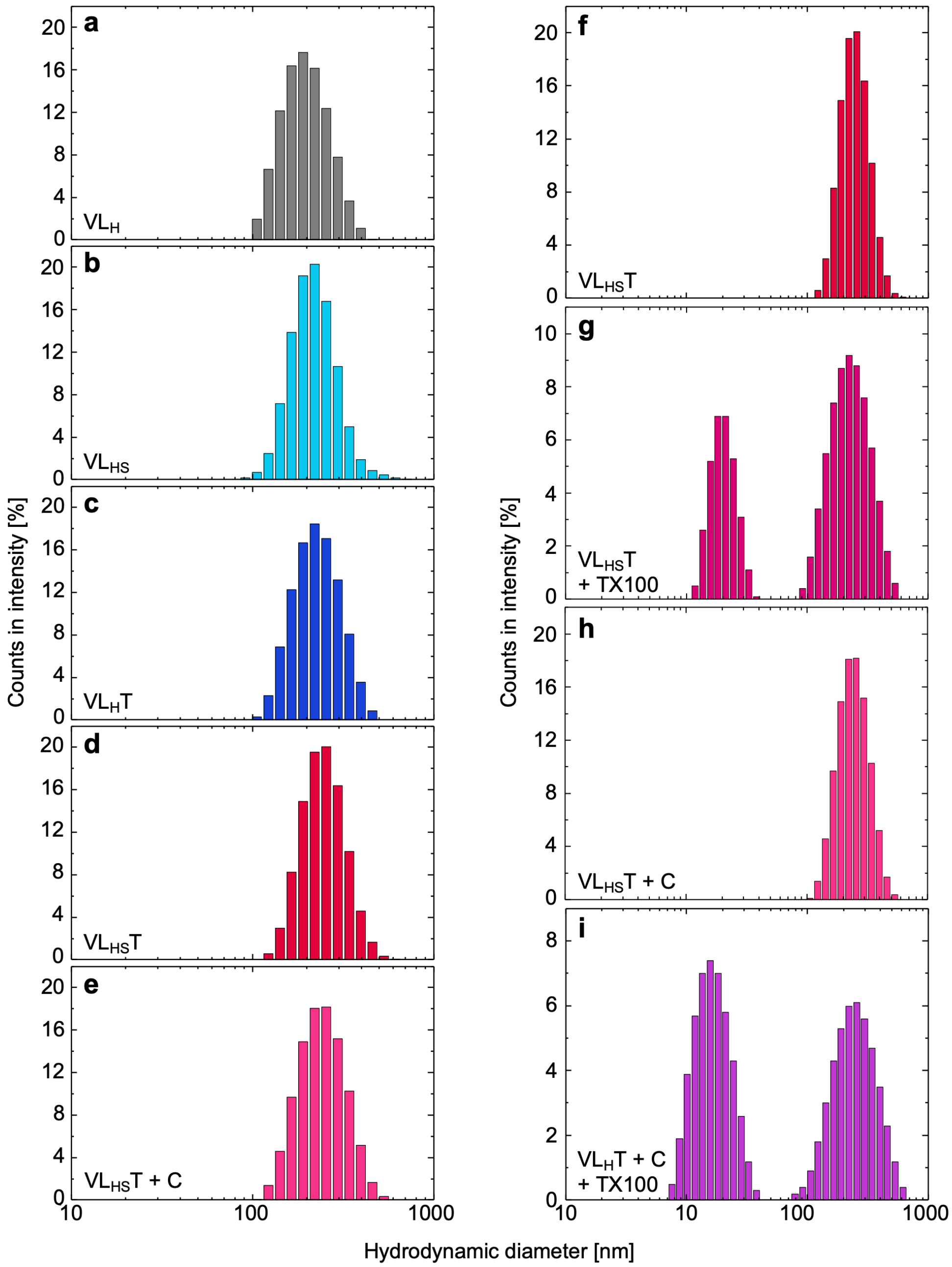

Figure S3. Size distribution (hydrodynamic diameters) obtained by dynamic light scattering for the DNA-liposome carriers at different assembly stages. (a) VL_H, (b) VL_{HS}, (c) VL_HT, (d) $VL_{HS}T$, (e) $VL_{HS}T$ + C. The addition of TX100 to VL_HT (f) and $VL_{HS}T$ (h) solubilizes the liposomes but leaves an extra peak assigned to DNA assemblies, (double peak in the DLS spectrum (g and i).

S5. Confocal Microscopy Imaging of DNA-Coated GUVs

Figure S4 shows the individual traces that were used to calculate the fluorescence recovery halftime and recovered fluorescence intensity. The curves were fitted using an exponential function with the equation

$$I(x) = C \cdot e^{\frac{-x}{\tau}} + I_{recov}$$

where *I(x)* is the current fluorescence intensity value after photobleaching, *C* a fitting constant, τ describes the time constant of the recovery trace, and I_{recov} the asymptote of the fluorescence intensity after recovery. The recovery halftime was calculated by solving the recovery function for x at $I\left(x_{halftime}\right) = 0.5 \cdot I_{recov}$.

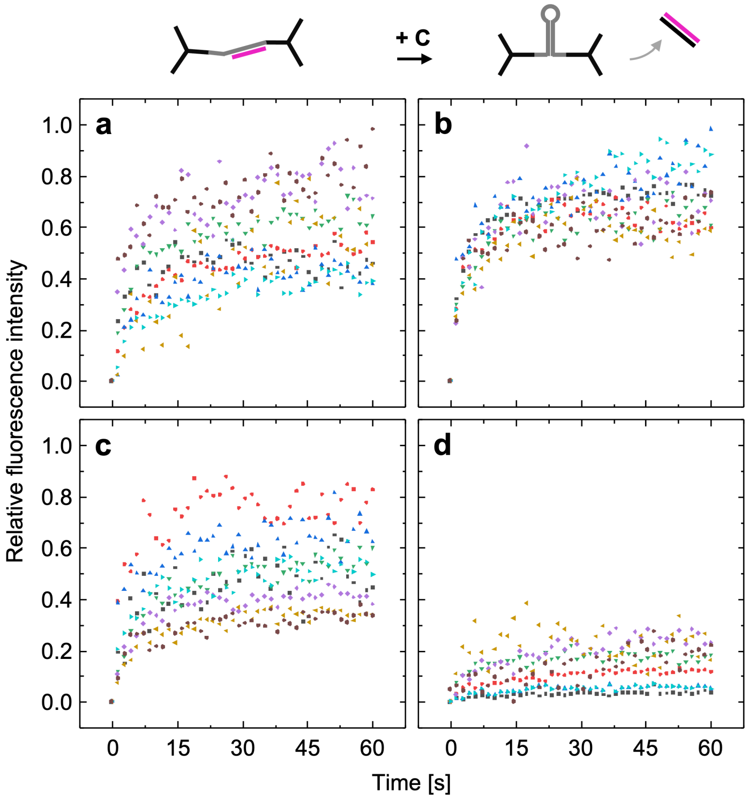

Figure S4. Individual FRAP measurements on GUVs before (a, c) and after (b, d) the addition of the displacement strand C (n = 8). Fluorescence recovery traces of the ATTO550-labelled S before (a) and after (b) the addition of C. Fluorescence recovery traces of the ATTO647N-labelled T before (c) and after (d) the addition of C. The calculated values for the recovery halftime and recovered fluorescence intensity can be found in the manuscript in Figure 4.

S6. Calcein Release Experiments

Figure S5 shows fluorescence intensity traces of passive and active calcein release as indicated. Most calcein passively leaks from V before the addition of C. After the addition of C, the largest escape of calcein is observed for pure liposomes (V + C) and shows a combination of passive leakage and release triggered by the change of the osmotic pressure by the addition of the additional volume containing C (present in all samples). Note that C does not otherwise interact with V. The second largest release of calcein is observed when the trigger-responsive carriers interact with C ($VL_{HS}T$ + C), whereas the addition of a non-hybridizing control C_c or the addition

of C to an inactive carrier design (VLT) leads to no significant fluorescence intensity increase. 1% TX100 was added to all samples for reference (maximum fluorescence intensity by destruction of the liposomes). The detergent disrupts the lipid bilayers and releases any residual calcein, which is consequently not quenched afterwards. To normalize the traces to the maximum fluorescence intensity, the following formula was applied:

$$I_{\%}(x) = \left(1 - \frac{I_{max,TX100} - I(x)}{I_{max,TX100} - I_{min}}\right) \cdot 100$$

$I_{max,TX100}$ denotes the maximum fluorescence intensity achieved by adding TX100. I_{min} describes the initial, minimum intensity at time t = 0.

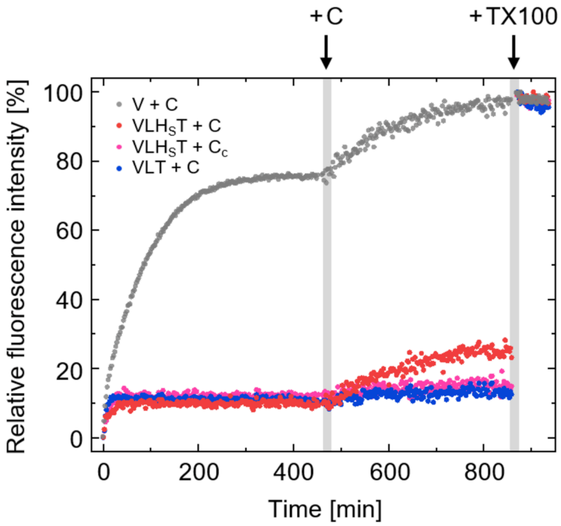

Figure S5. Fluorescence intensity increase corresponding to the passive calcein leakage, as well as release from DNA-liposome carriers as a result of the addition of C (representative traces).

S7. Liposome Preparation

The figure below depicts the liposome fabrication prior to the functionalization with the DNA structures schematically. The detailed fabrication procedure can be found in the methods section of the main text.

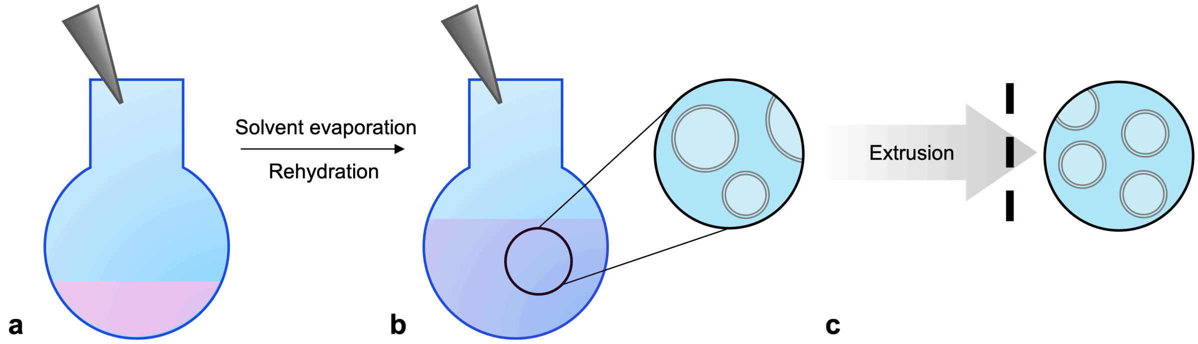

Figure S6. Schematic of the LUV preparation method. (a) The lipid suspension in chloroform is added to a round bottom flask. The chloroform is evaporated under vacuum, leaving a lipid film. (b) The lipid film is rehydrated in the desired buffer and the solution is sonicated. As a consequence, liposomes exhibiting a broad size distribution are generated. (c) The suspension is extruded through a membrane with a defined pore size to yield LUVs of a controlled average size and narrower distribution.

Supporting References

(1) Castro, C. E.; Kilchherr, F.; Kim, D.-N.; Shiao, E. L.; Wauer, T.; Wortmann, P.; Bathe, M.; Dietz, H. A Primer to Scaffolded DNA Origami. *Nat. Methods* **2011**, *8*, 221.

(2) Guilbaud, S.; Salomé, L.; Destainville, N.; Manghi, M.; Tardin, C. Dependence of DNA Persistence Length on Ionic Strength and Ion Type. *Phys. Rev. Lett.* **2019**, *122* (2). https://doi.org/10.1103/PhysRevLett.122.028102.

(3) Baumann, K. N.; Piantanida, L.; García-Nafría, J.; Sobota, D.; Voïtchovsky, K.; Knowles, T. P. J.; Hernández-Ainsa, S. Coating and Stabilization of Liposomes by Clathrin-Inspired DNA Self-Assembly. *ACS Nano* **2020**, *14* (2), 2316–2323. https://doi.org/10.1021/acsnano.9b09453.
